# Biomarker-guided antibiotic stewardship in suspected ventilator-associated pneumonia (VAPrapid2): a randomised controlled trial and process evaluation

**DOI:** 10.1016/S2213-2600(19)30367-4

**Published:** 2020-02

**Authors:** Thomas P Hellyer, Daniel F McAuley, Timothy S Walsh, Niall Anderson, Andrew Conway Morris, Suveer Singh, Paul Dark, Alistair I Roy, Gavin D Perkins, Ronan McMullan, Lydia M Emerson, Bronagh Blackwood, Stephen E Wright, Kallirroi Kefala, Cecilia M O'Kane, Simon V Baudouin, Ross L Paterson, Anthony J Rostron, Ashley Agus, Jonathan Bannard-Smith, Nicole M Robin, Ingeborg D Welters, Christopher Bassford, Bryan Yates, Craig Spencer, Shondipon K Laha, Jonathan Hulme, Stephen Bonner, Vanessa Linnett, Julian Sonksen, Tina Van Den Broeck, Gert Boschman, DW James Keenan, Jonathan Scott, A Joy Allen, Glenn Phair, Jennie Parker, Susan A Bowett, A John Simpson

**Affiliations:** a Translational and Clinical Research Institute, Newcastle University, Newcastle, UK; b National Institute for Health Research Newcastle In Vitro Diagnostics Cooperative, Newcastle University, Newcastle, UK; c Newcastle Clinical Trials Unit, Newcastle University, Newcastle, UK; d The Wellcome-Wolfson Centre for Experimental Medicine, Queen's University Belfast, Belfast, UK; e Regional Intensive Care Unit, The Royal Hospitals, Belfast, UK; f Northern Ireland Clinical Trials Unit, The Royal Hospitals, Belfast, UK; g Anaesthesia, Critical Care and Pain Medicine, University of Edinburgh, Queen's Medical Research Institute, Edinburgh, UK; h Intensive Care Unit, Royal Infirmary of Edinburgh, Edinburgh, UK; i Usher Institute, University of Edinburgh, Edinburgh, UK; j Division of Anaesthesia, Department of Medicine, University of Cambridge, Addenbrooke's Hospital, Cambridge, UK; k Department of Cancer and Surgery, Imperial College London, London, UK; l Division of Infection Immunity and Respiratory Medicine, Manchester National Institute for Health Research Biomedical Research Centre, University of Manchester, Manchester, UK; m Integrated Critical Care Unit, Sunderland Royal Hospital, City Hospitals Sunderland NHS Foundation Trust, Sunderland, UK; n Warwick Medical School, University of Warwick, Coventry, UK; o Intensive Care Unit, Heartlands Hospital, University Hospitals Birmingham NHS Foundation Trust, Birmingham, UK; p Integrated Critical Care Unit, Freeman Hospital, Newcastle upon Tyne Hospitals NHS Foundation Trust, Newcastle, UK; q Intensive Care Unit, Royal Victoria Infirmary, Newcastle upon Tyne Hospitals NHS Foundation Trust, Newcastle, UK; r Intensive Care Unit, Western General Hospital, Edinburgh, UK; s Intensive Care Unit, Manchester Royal Infirmary, Manchester University NHS Foundation Trust, Manchester, UK; t Intensive Care Unit, Countess of Chester NHS Foundation Trust, Chester, UK; u Institute of Ageing and Chronic Disease, University of Liverpool, Liverpool, UK; v Intensive Care Unit, University Hospital Coventry, University Hospitals Coventry and Warwickshire NHS Trust, Coventry, UK; w Intensive Care Unit, Northumbria Specialist Emergency Care Hospital, Cramlington, UK; x Intensive Care Unit, Preston Royal Hospital, Lancashire Teaching Hospitals NHS Foundation Trust, Preston, UK; y Intensive Care Unit, Sandwell General Hospital, Sandwell and West Birmingham Hospitals NHS Trust, West Bromwich, UK; z Intensive Care Unit, James Cook University Hospital, South Tees Hospitals NHS Foundation Trust, Middlesbrough, UK; aa Intensive Care Unit, Queen Elizabeth Hospital, Gateshead NHS Foundation Trust, Gateshead, UK; ab Intensive Care Unit, Russells Hall Hospital, Dudley Group NHS Foundation Trust, Dudley, UK; ac Becton Dickinson Biosciences Europe, Erembodegem, Belgium

## Abstract

**Background:**

Ventilator-associated pneumonia is the most common intensive care unit (ICU)-acquired infection, yet accurate diagnosis remains difficult, leading to overuse of antibiotics. Low concentrations of IL-1β and IL-8 in bronchoalveolar lavage fluid have been validated as effective markers for exclusion of ventilator-associated pneumonia. The VAPrapid2 trial aimed to determine whether measurement of bronchoalveolar lavage fluid IL-1β and IL-8 could effectively and safely improve antibiotic stewardship in patients with clinically suspected ventilator-associated pneumonia.

**Methods:**

VAPrapid2 was a multicentre, randomised controlled trial in patients admitted to 24 ICUs from 17 National Health Service hospital trusts across England, Scotland, and Northern Ireland. Patients were screened for eligibility and included if they were 18 years or older, intubated and mechanically ventilated for at least 48 h, and had suspected ventilator-associated pneumonia. Patients were randomly assigned (1:1) to biomarker-guided recommendation on antibiotics (intervention group) or routine use of antibiotics (control group) using a web-based randomisation service hosted by Newcastle Clinical Trials Unit. Patients were randomised using randomly permuted blocks of size four and six and stratified by site, with allocation concealment. Clinicians were masked to patient assignment for an initial period until biomarker results were reported. Bronchoalveolar lavage was done in all patients, with concentrations of IL-1β and IL-8 rapidly determined in bronchoalveolar lavage fluid from patients randomised to the biomarker-based antibiotic recommendation group. If concentrations were below a previously validated cutoff, clinicians were advised that ventilator-associated pneumonia was unlikely and to consider discontinuing antibiotics. Patients in the routine use of antibiotics group received antibiotics according to usual practice at sites. Microbiology was done on bronchoalveolar lavage fluid from all patients and ventilator-associated pneumonia was confirmed by at least 10^4^ colony forming units per mL of bronchoalveolar lavage fluid. The primary outcome was the distribution of antibiotic-free days in the 7 days following bronchoalveolar lavage. Data were analysed on an intention-to-treat basis, with an additional per-protocol analysis that excluded patients randomly assigned to the intervention group who defaulted to routine use of antibiotics because of failure to return an adequate biomarker result. An embedded process evaluation assessed factors influencing trial adoption, recruitment, and decision making. This study is registered with ISRCTN, ISRCTN65937227, and ClinicalTrials.gov, NCT01972425.

**Findings:**

Between Nov 6, 2013, and Sept 13, 2016, 360 patients were screened for inclusion in the study. 146 patients were ineligible, leaving 214 who were recruited to the study. Four patients were excluded before randomisation, meaning that 210 patients were randomly assigned to biomarker-guided recommendation on antibiotics (n=104) or routine use of antibiotics (n=106). One patient in the biomarker-guided recommendation group was withdrawn by the clinical team before bronchoscopy and so was excluded from the intention-to-treat analysis. We found no significant difference in the primary outcome of the distribution of antibiotic-free days in the 7 days following bronchoalveolar lavage in the intention-to-treat analysis (p=0·58). Bronchoalveolar lavage was associated with a small and transient increase in oxygen requirements. Established prescribing practices, reluctance for bronchoalveolar lavage, and dependence on a chain of trial-related procedures emerged as factors that impaired trial processes.

**Interpretation:**

Antibiotic use remains high in patients with suspected ventilator-associated pneumonia. Antibiotic stewardship was not improved by a rapid, highly sensitive rule-out test. Prescribing culture, rather than poor test performance, might explain this absence of effect.

**Funding:**

UK Department of Health and the Wellcome Trust.

**Research in context**

**Evidence before this study**

We searched Medline between Jan 1, 1996, and April 30, 2019, with the MeSH terms “Pneumonia”; “Pneumonia, bacterial”; “Pneumonia, Ventilator-Associated”; “Respiratory Tract Infections”; “Biomarkers”; “Protein Precursors”; and “Anti-bacterial Agents”. Although several trials investigated the role of procalcitonin in reducing antibiotic use in lower respiratory tract infections, to our knowledge, few trials have been done in patients with ventilator-associated pneumonia. A multicentre trial of a procalcitonin-guided intervention to discontinue antibiotics in patients with ventilator-associated pneumonia reported a significant improvement in antibiotic-free days at 28 days. However, the duration of antibiotics in both the intervention and the control groups of the trial were longer than the 8-day duration recommended in international guidelines. A further single-centre trial used a combination of the Clinical Pulmonary Infection Score and procalcitonin to guide antibiotic discontinuation in patients who had already completed 7 days of antibiotic therapy. Although patients in the procalcitonin group had more antibiotic-free days at 28 days versus the control group, the duration of antibiotics in both groups was longer than 8 days. These studies focused on discontinuation of antibiotics once empirical treatment was established. To our knowledge, there are no published trials in which antibiotic stewardship is based on early exclusion of ventilator-associated pneumonia.

**Added value of this study**

To our knowledge, VAPrapid2 is the first trial to use a validated biomarker in a cohort of patients with clinically suspected ventilator-associated pneumonia, with an aim to determine whether early exclusion of ventilator-associated pneumonia could improve antibiotic stewardship. Furthermore, our trial included a process evaluation that aimed to understand clinical behaviours and implementation of the trial protocol. This trial showed that, although the biomarker test could accurately exclude ventilator-associated pneumonia, the trial recommendation regarding antibiotic discontinuation was seldom followed by clinicians, resulting in no difference in antibiotic use between the intervention and control groups. The results of this trial highlight entrenched behaviours in antibiotic prescribing practice and barriers to adopting new, unfamiliar technologies.

**Implications of all the available evidence**

Previous trials of procalcitonin have influenced the duration of antibiotic treatment in patients with ventilator-associated pneumonia. However, most patients with suspected ventilator-associated pneumonia do not actually have it, subjecting them to unnecessary antibiotic treatment while the true cause of respiratory compromise potentially goes untreated. Avoiding antibiotic use in such patients remains an important goal for antibiotic stewardship in intensive care units. The VAPrapid2 trial showed no influence on antibiotic prescribing practices in this patient group. Future studies should differentiate suspected from confirmed ventilator-associated pneumonia, aim to reduce antibiotics in patients who do not have confirmed infection, and dissect complex mechanisms that influence prescribing practices.

## Introduction

Ventilator-associated pneumonia is the most common infection acquired in intensive care units (ICUs),^1^ and is associated with substantial mortality, particularly in the ageing ICU population.^2^ Broad-spectrum antibiotic use is recommended in suspected ventilator-associated pneumonia.^3^, ^4^ However, diagnosis of this infection remains notoriously difficult, and pulmonary infection is typically confirmed in only 20–60% of suspected cases.^5^ Consequently, antibiotics are overused for suspected ventilator-associated pneumonia, potentially exposing patients to adverse effects, detracting from alternative causes of respiratory compromise, increasing costs, and driving emergence of antimicrobial resistance.^5^

Point prevalence studies suggest that 70% of patients in the ICU receive antibiotics.^6^ The association between increased antibiotic use and emergence of antimicrobial resistance in ICUs is well established.^7^ In the setting of hospital-acquired pneumonia, adherence to guidelines that promote broad-spectrum empirical antibiotics has been associated with adverse outcomes.^8^ This background has driven a need to rationalise antibiotic prescribing in ICUs.

Rapid diagnostic tests with the capacity to rule out ventilator-associated pneumonia might present early opportunities to optimise antibiotic prescription and decrease antibiotic use. Among protein-based biomarkers, only a combination of low IL-1β and IL-8 concentrations in bronchoalveolar lavage fluid has been validated in a multicentre setting in suspected ventilator-associated pneumonia.^9^, ^10^

The VAPrapid2 trial aimed to determine whether measurement of bronchoalveolar lavage fluid IL-1β and IL-8 could improve antibiotic stewardship without compromising patient safety in suspected ventilator-associated pneumonia. In keeping with expert guidance on analysis of complex interventions,^11^ a process evaluation study was embedded in this trial.

## Methods

### Study design and participants

VAPrapid2 was a multicentre, randomised controlled trial in patients admitted to the ICU with suspected ventilator-associated pneumonia. The trial was done in 24 ICUs from 17 National Health Service (NHS) hospital trusts across England, Scotland, and Northern Ireland.

Patients were screened for eligibility on weekdays and included if they were aged 18 years or older, intubated and mechanically ventilated for at least 48 h, and had suspected ventilator-associated pneumonia. Criteria for suspected ventilator-associated pneumonia were new or worsening chest radiographic (x-ray or chest CT) alveolar changes plus at least two of the following: body temperature less than 35°C or greater than 38°C, white cell count less than 4 × 10^9^/L or greater than 11 × 10^9^/L, and purulent tracheal secretions.^5^ Additionally, clinicians had to consider eligible patients unlikely to have extrapulmonary infection requiring antibiotic treatment (ie, early discontinuation of antibiotics would be appropriate if ventilator-associated pneumonia was confidently excluded).

Patients were excluded if they fulfilled the criteria predicting poor tolerance of bronchoscopy and bronchoalveolar lavage: PaO_2_ less than 8 kPa on FiO_2_ greater than 0·7, positive end-expiratory pressure greater than 15 cmH_2_O, peak airway pressure greater than 35 cmH_2_O, heart rate greater than 140 beats per minute, mean arterial pressure less than 65 mm Hg, bleeding diathesis (platelet count <20 × 10^9^/L or international normalised ratio >3), intracranial pressure greater than 20 mm Hg, and ICU consultant considered bronchoscopy and bronchoalveolar lavage to be unsafe for the patient.

The research protocol was approved by the England and Northern Ireland (13/LO/065) and Scotland (13/SS/0074) National Research Ethics Service committees, and the trial protocol has been published previously.^12^ Patients or their relatives or representatives gave written informed consent for inclusion in the study.

### Randomisation and masking

Patients were randomly assigned (1:1) to biomarker-guided recommendation on antibiotics (intervention group) or routine use of antibiotics (control group) using a web-based randomisation service hosted by Newcastle Clinical Trials Unit (NCTU). Randomisation was triggered by the technician receiving each patient's bronchoalveolar lavage fluid sample. The randomisation sequence was generated by the trial statistician using Sealed Envelope. Patients were randomised using randomly permuted blocks of size four and six and stratified by site, with allocation concealment. Participants underwent the same clinical procedures up to the point biomarker results were returned to the clinical service for the intervention group. Therefore, there was an initial period of double-blinding until test results were communicated to clinicians. As such, clinicians and research nurses were masked until the biomarker results became available.

### Procedures

Investigators were asked to record a clinical opinion on the pre-test probability of ventilator-associated pneumonia in randomly assigned patients—high, medium, or low. A protocolised bronchoscopy and bronchoalveolar lavage was arranged for all randomly assigned patients using a 120 mL lavage with 0·9% saline.^10^ Samples were transported at 4°C to one of six testing laboratories (appendix p 5), with a transport time of up to 1·5 h.

IL-1β and IL-8 in bronchoalveolar lavage fluid were measured by cytometric bead array using Accuri C6 flow cytometers (Becton Dickinson Biosciences; San Jose, CA, USA). IL-1β and IL-8 concentrations in bronchoalveolar lavage fluid were entered into a previously derived equation for the exclusion of ventilator-associated pneumonia,^10^ and an automated calculation was made available to the laboratory staff processing bronchoalveolar lavage fluid samples. Instructions were communicated to clinicians by telephone immediately after results became available. For patients randomly assigned to the biomarker-guided group, the instruction relayed to clinicians was either, “Biomarker result above cutoff. Ventilator-associated pneumonia cannot be excluded, consider continuing antibiotics,” or “Biomarker result below cutoff. The negative predictive value is 1 and ventilator-associated pneumonia is very unlikely. Consider discontinuation of antibiotics.” For patients randomly assigned to routine use of antibiotics the instruction given to clinicians was, “Patient in routine use of antibiotics group.” If assays did not meet internal quality control criteria, clinicians were advised to default to routine care. The median negative predictive value previously calculated for the combination of IL-1β and IL-8 was 1·0 (95% CI 0·92–1·0).^10^

We defined confirmed ventilator-associated pneumonia as growth of a potentially pathogenic organism of at least 10^4^ colony forming units (CFU) per mL of bronchoalveolar lavage fluid.^13^ Microbiology testing was done in accredited NHS or Public Health England microbiology laboratories. Standard operating procedures for semiquantitative culture were done in accordance with the 2012 UK Standards for Microbiology Investigation, issued by the Health Protection Agency. This strategy allowed samples to be quantified as having no growth, 1–10 CFU/mL, 10–10^2^ CFU/mL, 10^2^–10^3^ CFU/mL, 10^3^–10^4^ CFU/mL, 10^4^–10^5^ CFU/mL, and so on, allowing simple and clear demarcation of bacteria grown at 10^4^ CFU/mL or more (ventilator-associated pneumonia) and less than 10^4^ CFU/mL.

Investigators visited all ICUs before recruitment commenced, providing educational sessions on the diagnostic performance of the biomarkers and on the trial intervention. Key components of trial design were reinforced through regular communication. Additional training was done in testing laboratories with respect to laboratory processes and biomarker measurement. Before the trial commenced, clinicians were again made aware of the biomarker test and were encouraged to follow the biomarker-guided recommendations. However, antibiotic use decisions were not mandated and were at clinicians' discretion.

A process evaluation was done with interviews of clinical staff and research staff (eg, site principal investigator, consultants, research nurses, and ward manager) in the following three phases: pre-trial (in month 1 of sites joining the trial; exploring routine diagnosis and management of ventilator-associated pneumonia), mid-trial (once a site was involved in the trial for at least 1 year; exploring intervention quality, attitudes to the trial, and barriers or facilitators to successful trial delivery), and late-trial, with purposive sampling of nine sites based on pre-trial and mid-trial results (in the final 3 months of the intervention period in June to Augsust, 2016; exploring local factors determining recruitment). Interviews were done by LME. Further details are in the appendix (p 4).

### Outcomes

The primary outcome was the distribution of antibiotic-free days in the 7 days following bronchoalveolar lavage. Antibiotic-free days were handled as an integer, with patients classified in one of eight categories (0–7 antibiotic-free days, inclusive).

Predefined secondary outcomes were antibiotic-free days at days 14 and 28, antibiotic days at days 7, 14, and 28, ventilator-free days at 28 days, 28-day mortality and ICU mortality, sequential organ failure assessment (SOFA) score at days 3, 7 and 14, duration of critical care (level 2 and level 3 care) and hospital stay, antibiotic-associated infections (*Clostridium difficile* and meticillin-resistant *Staphylococcus aureus*) up to hospital discharge, death, or 56 days, antibiotic-resistant pathogens (resistant to two or more antibiotics) cultured up to hospital discharge, death, or 56 days, and health-care resource use calculated from length of critical care and hospital stay up to discharge, death, or 56 days. When considering outcomes at days 7, 14, and 28, antibiotics refers to all antibiotics given for treatment of infection; prophylactic antibiotics were not considered.

Since adverse clinical events are common in ICUs, the trial protocol mandated reporting of adverse events within 2 h of bronchoscopy. Clinical team members reported any further events after 2 h if they were considered clinically significant or related to the trial.

### Statistical analysis

Full statistical methods were outlined in a Statistical Analysis Plan before the close of recruitment. Sample size was based on the change in frequency distribution of antibiotic-free days in the 7 days following bronchoalveolar lavage. Models of change in distribution are outlined in the trial protocol.^12^ We deemed effect sizes in the region 0·07–0·08 to be of a clinically relevant magnitude. These effect sizes represent an approximate change in median antibiotic-free days from 0 (IQR 0·0–2·5) to 1·5 (0·0–3·5). Therefore, we proposed a recruitment target of 90 patients per group, with an α of 0·05 and β of 0·20. Allowing for attrition of 14·3%, the target sample size was 210 patients. The primary analysis was done on the intention-to-treat population. We analysed the primary outcome by χ^2^ test on a 2 × 8 table of trial group versus antibiotic-free days. Sensitivity analyses were done using a discrete-time Cox proportional hazards model with centre and randomisation group as covariates, censored for death or end of follow-up at 7 days. We did a further sensitivity analysis, redefining antibiotic-free days as zero if death occurred within 7 days, as a more conservative approach.

We analysed secondary outcomes using Cox proportional hazards models, logistic regression, linear regression, or Poisson regression as appropriate (see appendix p 4). Planned subgroup analyses included a per-protocol analysis, clinician assessment of likelihood of ventilator-associated pneumonia, and admission category (medical, surgical caused by trauma or head injury, and other surgical). We excluded patients randomly assigned to the intervention group who defaulted to routine use of antibiotics from the per-protocol analysis because they did not return a biomarker result.

We assessed the prevalence of missing data during a masked review after database lock, which was judged to be of sufficiently low frequency as to not require imputation for all variables. However, as prespecified in the Statistical Analysis Plan, SOFA scores were based on the last evaluable score. Unadjusted CIs and p values are reported for multiplicity.

The Newcastle upon Tyne Hospitals NHS Foundation Trust acted as sponsor for the trial. Clinical trial management was provided by the NCTU. An independent data monitoring and safety committee oversaw the trial (appendix p 3).

Analyses were done with R version 3.3.2, with the addition of the discSurv package (version 1.3.4). This study is registered with ISRCTN, ISRCTN65937227, and ClinicalTrials.gov, NCT01972425.

### Role of the funding source

The funder of the study had no role in study design, data collection, data analysis, data interpretation, or writing of the report. The corresponding author had full access to all the data in the study and had final responsibility for the decision to submit for publication.

## Results

Between Nov 6, 2013, and Sept 13, 2016, 360 patients were screened for inclusion in the study. 146 patients were ineligible, leaving 214 who were recruited to the study. Four patients were excluded before randomisation, meaning that 210 patients were randomly assigned to biomarker-guided recommendation on antibiotics (n=104) or routine use of antibiotics (n=106; figure). One patient in the biomarker-guided recommendation group was withdrawn by the clinical team once baseline data were collected but before bronchoscopy, so was excluded from the intention-to-treat analysis.

**Figure:**
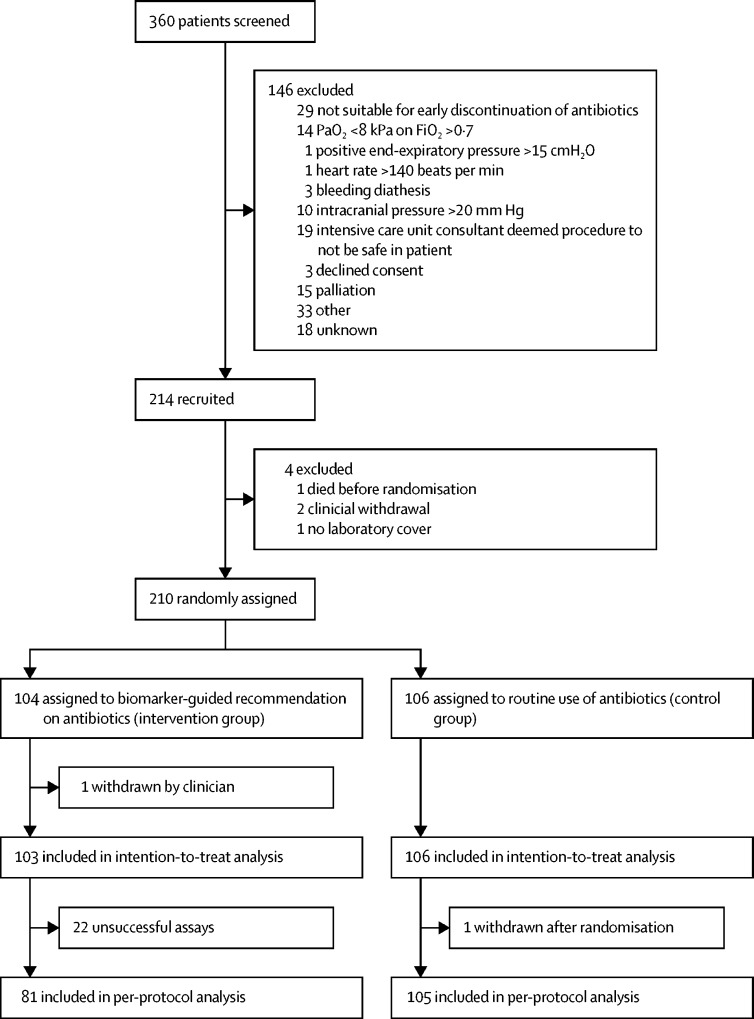
Trial profile

Ventilator-associated pneumonia was confirmed in more patients in the intervention group than in the control group (table 1). The most commonly isolated pathogen in bronchoalveolar lavage fluid was *S aureus* (table 2). Clinician pre-test suspicion of ventilator-associated pneumonia was high in most patients in the study (table 1). The biomarker assay had a negative likelihood ratio of 0·09 (95% CI 0·01–0·68; for more detail on test performance see appendix p 6). The mean time from bronchoalveolar lavage to reporting results was 8 h and 10 min (SD 2 h 31 min).

**Table 1:** Baseline characteristics

		**Biomarker-guided recommendation on antibiotics group (n=103)**	**Routine use of antibiotics group (n=106)**
Age (years)		57·5 (16·1)	56·2 (15·9)
Sex			
	Female	37 (36%)	39 (37%)
	Male	66 (64%)	67 (63%)
APACHE II score		18·6 (8·3)	16·6 (6·8)
Total SOFA score		5·2 (2·5)	4·9 (2·8)
	SOFA respiratory	2·6 (0·9)	2·4 (1·0)
	SOFA renal	0·4 (0·8)	0·5 (1·0)
	SOFA hepatic	0·3 (0·6)	0·4 (0·8)
	SOFA cardiovascular	1·6 (1·6)	1·3 (1·6)
	SOFA haematological	0·3 (0·6)	0·4 (0·8)
Functional comorbidity index score		1·3 (1·4)	1·4 (1·2)
Medical admission		59 (57%)	70 (66%)
Surgical admission		44 (43%)	36 (34%)
Admission category			
	Respiratory	18 (17%)	24 (23%)
	Gastrointestinal or liver	7 (7%)	10 (9%)
	Cardiovascular	17 (17%)	9 (8%)
	Trauma	23 (22%)	23 (22%)
	Sepsis	4 (4%)	4 (4%)
	Obstetrics and gynaecology	0	1 (1%)
	Neurological (non-trauma)	20 (19%)	21 (20%)
	Other	14 (14%)	14 (13%)
Clinician pre-test suspicion of ventilator-associated pneumonia*			
	Low	10 (10%)	7 (7%)
	Medium	33 (32%)	51 (48%)
	High	59 (57%)	48 (45%)
Number of days in ICU before bronchoalveolar lavage†		10·2 (8·7)	10·6 (10·4)
Confirmed ventilator-associated pneumonia‡		38 (37%)	32 (30%)
Acute respiratory distress syndrome§			
	Mild (26·7–40·0 kPa)	11 (11%)	11 (10%)
	Moderate (13·3–<26·7 kPa)	16 (16%)	11 (10%)
	Severe (<13·3 kPa)	1 (1%)	5 (5%)
Vasopressors†		38 (37%)	37 (35%)
Renal replacement therapy		8 (8%)	4 (4%)
Use of corticosteroids		17 (17%)	15 (14%)
Receiving antibiotics at randomisation		83 (81%)	87 (82%)
Temperature (°C)*		37·4 (1·0)	37·7 (0·9)
White cell count (× 10^9^/L)		14·4 (5·4)	15·5 (7·2)
C-reactive protein (mg/L)¶		153·0 (97·9)	157·6 (107·3)
Positive end-expiratory pressure (cm H_2_O)‖		7·5 (2·9)	7·8 (2·6)
Peak airway pressure (cm H_2_O)**		20·5 (6·4)	21·3 (7·1)
PaO_2_:FiO_2_ (kPa)††		29·1 (20·4–39·7)	25·3 (18·5–36·0)

Data are mean (SD), n (%), or median (IQR). APACHE=acute physiology and chronic health evaluation. SOFA=sequential organ failure assessment. ICU=intensive care unit.

* One patient from the biomarker-guided group missing.

† Three patients (one from the biomarker-guided group) missing.

‡ One patient from the routine use of antibiotics group missing.

§ Two patients (one from the biomarker-guided group) missing.

¶ 85 patients (43 from the biomarker-guided group) missing.

‖ Two patients from the biomarker-guided group missing.

** 23 patients (eight from the biomarker-guided group) missing.

†† Seven patients (three from the biomarker-guided group) missing.

**Table 2:** Microorganisms isolated in bronchoalveolar lavage fluid

	**Ventilator-associated pneumonia (≥10^4^ CFU/mL; n=70)**	**Non-ventilator-associated pneumonia (<10^4^ CFU/mL; n=139)**
*Staphylococcus aureus*	26	21*
*Haemophilus* spp	12	4
*Escherichia coli*	9	6
*Pseudomonas aeruginosa*	6	4
*Klebsiella* spp	5	15
*Proteus mirabilis*	5	3
*Stenotrophomonas maltophilia*	3	5
*Streptococcus pneumoniae*	3	1
Yeasts	3	13
*Candida* spp	2	17
*Citrobacter* spp	2	2
*Enterobacter* spp	2	8
*Serratia marcescens*	2	3
*Staphylococcus* spp	2	6
*Streptococcus* spp	2	2
*Acinetobacter* spp	1	2
Anaerobes	1	0
Coliform	1	3
*Corynebacterium* spp	1	0
*Enterococcus* spp	1	1
Gram-positive cocci	1	2
Moraxella catarrhalis	1	0
*Prevotella* spp	1	0
Aspergillus fumigatus	0	4
Burkholderia vietnamiensis	0	1
Morganella morganii	0	2
*Neisseria* spp	0	1

Data are n of isolates (in some bronchoalveolar lavage samples, more than one organism was isolated). 68 patients in the non-ventilator-associated pneumonia column had no growth. CFU=colony-forming units.

* In one case the organism isolated was meticillin-resistant *Staphylococcus aureus*.

We found no significant difference in the primary outcome of the distribution of antibiotic-free days in the 7 days following bronchoalveolar lavage, either in the intention-to-treat (p=0·58; table 3; appendix p 7) or per-protocol analyses (p=0·28, table 3). In the biomarker-guided group the IL-1β and IL-8 result was high in 64 patients and low in 17 patients. In these 17 patients, the recommendation to discontinue antibiotics was followed in four (24%) patients, and a false negative result was obtained in one (6%) patient. Microbiological details relating to bronchoalveolar lavage fluid from the 17 patients with a low IL-1β and IL-8 result (ie, those eligible for antibiotic discontinuation) are shown in the appendix (p 8).

**Table 3:** Antibiotic-free days for the intention-to-treat and per-protocol analyses

	**0**	**1**	**2**	**3**	**4**	**5**	**6**	**7**
**Intention-to-treat analysis**								
Biomarker-guided recommendation on antibiotics group (n=102)	50 (49%)	13 (13%)	7 (7%)	10 (10%)	7 (7%)	3 (3%)	4 (4%)	8 (8%)
Routine use of antibiotics group (n=105)	40 (38%)	14 (13%)	13 (12%)	18 (17%)	6 (6%)	4 (4%)	4 (4%)	6 (6%)
**Per-protocol analysis**								
Biomarker-guided recommendation on antibiotics group (n=80)	42 (53%)	10 (13%)	7 (9%)	5 (6%)	6 (8%)	1 (1%)	3 (4%)	6 (8%)
Routine use of antibiotics group (n=105)	40 (38%)	14 (13%)	13 (12%)	18 (17%)	6 (6%)	4 (4%)	4 (4%)	6 (6%)

Data are n (%).

We observed no significant differences between the groups for all other secondary outcomes (table 4). Results for subgroup analyses, per-protocol analyses, and antibiotic-resistant infections are shown in the appendix (pp 10–17). Our two sensitivity analyses—one treating death as equivalent to zero antibiotic-free days and the other censoring at death in a discrete-time Cox model—revealed no difference in the primary outcome in the intention-to-treat population (appendix p 9).

**Table 4:** Secondary outcome measures

	**Biomarker-guided recommendation on antibiotics group (n=103)**	**Routine use of antibiotics group (n=106)**	Effect size*(95% CI)
Antibiotic days (7 days post-bronchoalveolar lavage)†	6 (4–7)	6 (4–7)	HR 0·84 (0·63 to 1·12)
Antibiotic days (14 days post-bronchoalveolar lavage)‡	8 (6–12)	8 (5–11)	HR 0·94 (0·69 to 1·28)
Antibiotic days (28 days post-bronchoalveolar lavage)§	11 (7–15)	10 (5–15)	HR 0·90 (0·65 to 1·25)
Antibiotic-free days (14 days post-bronchoalveolar lavage)‡	6 (2–8)	6 (3–9)	HR 1·13 (0·83 to 1·54)
Antibiotic-free days (28 days post-bronchoalveolar lavage)§	17 (13–21)	18 (13–23)	HR 1·01 (0·73 to 1·40)
Days of critical care stay¶	14 (8–23)	14 (8–22)	HR 1·00 (0·73 to 1·39)
Days of hospital stay‖	27 (16–58)	28 (12–50)	HR 0·83 (0·60 to 1·15)
Days of level 3 (intensive care) stay¶	10 (5–18)	10 (6–17)	HR 1·05 (0·76 to 1·45)
Days of level 2 (high dependency) stay**	3 (1–8)	4 (1–7)	HR 1·05 (0·74 to 1·48)
Mortality at 28 days††	28 (27%)	21 (20%)	OR 1·52 (0·78 to 2·98)
ICU mortality	25 (24%)	20 (19%)	OR 1·35 (0·68 to 2·71)
Presence of antibiotic-associated infections to hospital discharge, death, or 56 days††	6 (6%)	7 (7%)	OR 0·86 (0·26 to 2·71)
SOFA score at 3 days‡‡	4·3 (2·6)	4·4 (2·7)	−0·18 (−0·87 to 0·52)
SOFA score at 7 days§§	4·0 (2·8)	4·1 (2·7)	−0·13 (−0·85 to 0·59)
SOFA score at 14 days§§	3·7 (2·8)	3·6 (3·0)	0·00 (−0·76 to 0·77)
Ventilator-free days (at 28 days)	11 (0–19)	9 (0–19)	RR 1·03 (0·94 to 1·12)
Number of antibiotic-resistant pathogens to hospital discharge, death or 56 days††	0 (0–0)	0 (0–0)	RR 1·71 (1·16 to 2·57)
Number of pathogens (outlier excluded)	0 (0–0)	0 (0–0)	RR 1·36 (0·90 to 2·08)

Data are median (IQR), n (%), or mean (SD), unless otherwise indicated. The last row of the table excludes a single observation in the biomarker-guided group recorded as having multiple pathogens (more than twice any other patient). HR=hazard ratio. ICU=intensive car e unit. OR=odds ratio. SOFA=sequential organ failure assessment. RR=risk ratio.

* Cox proportional hazards presented as HR, logistic regression presented as OR, linear regression presented as mean difference, and Poisson regression presented as RR.

† Two patients (one from the biomarker-guided group) missing.

‡ 21 patients (11 from the biomarker-guided group) missing.

§ 34 patients (14 from the biomarker-guided group) missing.

¶ Six patients (three from the biomarker-guided group) missing.

‖ Five patients (two from the biomarker-guided group) missing.

** 61 patients (30 from the biomarker-guided group) missing.

†† One patient from the biomarker-guided group missing.

‡‡ 12 patients (five from the biomarker-guided group) missing.

§§ Eight patients (three from the biomarker-guided group) missing.

Zero antibiotic-free days was the most frequent prescribing outcome (table 3). Median antibiotic days at day 7 were 6 (IQR 4–7) in both groups (hazard ratio [HR] 0·84, 95% CI 0·63–1·12; table 4). We found no between-group differences in antibiotic-free days at 14 days or 28 days (table 4). Reported indications for antibiotics are described in the appendix (pp 18–19).

Numbers of patients with one or more reported adverse events or serious adverse events are shown in table 5. Details of adverse events and serious adverse events are shown in the appendix (p 20). Bronchoalveolar lavage was associated with a small, transient increase in oxygen requirements (appendix p 21).

**Table 5:** Patients with reported adverse events or serious adverse events

		**Biomarker-guided recommendation on antibiotics group (n=103)**	**Routine use of antibiotics group (n=106)**
Adverse event or serious adverse event reported		43 (42%)	37 (35%)
	Adverse event recorded	39 (91%)	35 (95%)
	Serious adverse event recorded	4 (9%)	2 (5%)

Data are number of patients (%); multiple events were reported in some patients.

In the 13 patients in whom the discontinuation recommendation was not followed, our process evaluation suggested that perceived ventilator-associated pneumonia or hospital-acquired pneumonia was the most common reason for antibiotic use. The process evaluation identified two broad potentially negative influences on recruitment to the trial and compliance with the trial intervention. The first of these influences suggested that the chain involving identification of potential participants, preparation for bronchoalveolar lavage, laboratory processing, and a clinician making a judgement on the basis of the recommendation, introduced many opportunities for deviation from the model. A breakdown in this sequence, at any stage, negatively affected site performance and implementation of the intervention. Second, we identified a pattern such that low recruitment by units appeared to correspond with less use of bronchoalveolar lavage in the diagnosis of ventilator-associated pneumonia outside the trial, a culture of not actively de-escalating antibiotics, and the absence of so-called trial champions (designated as having a particular interest in promoting and delivering the trial within a given unit). These same units also described a greater perception of risk for bronchoalveolar lavage (favouring less invasive methods) and for discontinuing antibiotics (favouring antibiotic use as the perceived lower-risk approach). For further details of the process evaluation see the appendix (pp 22–24).

## Discussion

In the VAPrapid2 trial, a validated test with good rule-out characteristics for ventilator-associated pneumonia did not reduce antibiotic use or improve any of our other investigated clinical outcomes. To our knowledge, this is the first trial to use biomarkers to exclude ventilator-associated pneumonia to increase confidence in early discontinuation of empirical antibiotics. Previous studies have shown proof of principle for modest antibiotic reduction in suspected ventilator-associated pneumonia using discontinuation rules.^14^, ^15^ Serum procalcitonin has been studied widely in the ICU (outside the specific context of ventilator-associated pneumonia) and this approach showed varying success for safely adjusting antibiotic use.^16^, ^17^, ^18^, ^19^, ^20^ However, procalcitonin is ineffective for the exclusion of ventilator-associated pneumonia,^21^, ^22^ which was the focus of our approach. Inconsistent effects of procalcitonin on antibiotic use have also been described for lower respiratory tract infection outside the ICU.^23^, ^24^ Enthusiasm for a procalcitonin strategy in the ICU is offset by high non-compliance with procalcitonin guidance in general, and durations of procalcitonin-guided antibiotic use in ventilator-associated pneumonia that exceed the widely accepted standard of 8 days.^3^, ^4^, ^15^, ^25^ Given that confirmation of ventilator-associated pneumonia is low in cases in which it is suspected,^5^ we reasoned that persuasive early evidence for the absence of ventilator-associated pneumonia might have a greater effect on antibiotic duration. Proof of concept for this general strategy had been provided by a single-centre study that used preliminary microbiology culture results to stop antibiotics around 1 day after bronchoalveolar lavage.^26^

IL-1β and IL-8 fulfill the widely accepted criteria for a good exclusion test because they have a negative likelihood ratio of less than 0·1.^27^ This second, independent validation of their performance in 24 ICUs under real-life conditions supports their diagnostic utility. This study yielded opportunities to discontinue antibiotics, but advice was only followed in four (24%) of 17 cases.

To our knowledge, this trial is among the first in critical care to embed a process evaluation, with the aim of understanding behaviours, in accordance with expert guidance.^11^ Our data suggest that the observed absence of effect was more likely to be explained by clinicians' behaviour than by poor test performance. Before the trial commenced, the negative predictive value of the test was estimated at 1·0 (95% CI 0·92–1·00),^10^ therefore, the lower confidence limit could have affected clinicians' confidence. However, the process evaluation did not provide evidence to support this theory. The process evaluation suggested that deeply entrenched prescribing characteristics probably underlie the lack of effect, as described in other settings.^28^, ^29^ We attempted to mitigate non-compliance by including ICUs committed to the principle of accepting a recommendation to stop antibiotics. However, dissociation between intention and action in prescribing has been previously described.^30^, ^31^, ^32^

Inherent concern around failing to treat potential ventilator-associated pneumonia could have influenced prescribing, with fewer antibiotic-free days when pre-test probability was high. Beliefs around assumed efficacy and safety of antibiotics shape prescribing in emergency departments and presumably the motivation to avoid harm is enhanced in suspected ventilator-associated pneumonia in the ICU.^28^

In keeping with the broad spectrum of factors that determine successful introduction of complex diagnostic interventions,^11^, ^33^ our process evaluation provided valuable and unexpected insights into influences that might affect delivery of a trial. The process evaluation suggested that recruitment often did not proceed because of identification of restricted availability of bronchoalveolar lavage or laboratory processing on the day, or because clinicians habitually preferred to complete antibiotic courses (thereby precluding enrolment). However, the main barrier to recruitment was clinician scepticism around the additional diagnostic value and safety of bronchoalveolar lavage. The role of bronchoalveolar lavage in the diagnosis of ventilator-associated pneumonia remains contentious.^34^, ^35^ Bronchoalveolar lavage is not a gold standard diagnostic for ventilator-associated pneumonia—simultaneous histological analysis and microbiological culture of alveolar tissue would give ideal diagnostic precision, but is neither practical nor ethical. We considered bronchoalveolar lavage the most pragmatic and accurate alternative, while recognising it to be an imperfect reference standard.^5^ Considerable lack of familiarity with bronchoalveolar lavage in ICUs has been highlighted previously.^36^ The validation of test accuracy suggests that protocolised bronchoalveolar lavage was done to a high and uniform standard in this study. Furthermore, bronchoalveolar lavage was generally safe, although was associated with a small, transient increase in oxygen requirements, which has been noted elsewhere.^37^ Finally, the process evaluation strongly suggested that permanent research champions and ICU-trained research nurses devoted to the trial enhanced all aspects of recruitment and trial delivery. In units without research champions, attrition of recruitment over time was pronounced and self-perpetuating.

There are several limitations to this study. There was a higher than expected number of assay failures early in the trial. Not all centres had the assay on site, although all had access within around 90 min of sample collection. The mean time taken to return test results from bronchoalveolar lavage was more than 8 h. The study design left the decision to prescribe antibiotics with clinicians, rather than mandating stopping in accordance with test recommendations. Sites did not have a dedicated investigator who was responsible for recommending discontinuation of antibiotics. We did not systematically collect data on sites' pre-existing antibiotic stewardship policies or use of rapid diagnostics for infection, and we acknowledge the subjectivity of pre-test probability on the basis of clinician judgement. Although extrapulmonary infection was considered unlikely in eligible patients in our study, we cannot be certain that sources of infection outside the lung parenchyma (eg, pleural infection or subphrenic abscess) were absent. We did not collect clinical pulmonary infection scores^38^ or ventilator-associated pneumonia severity scores,^39^, ^40^ and we did not ascertain why patients who were taking corticosteroids had been prescribed these.

Whether fungi and various bacteria traditionally considered commensals can be considered a cause of ventilator-associated pneumonia when isolated from bronchoalveolar lavage at 10^4^ CFU/mL or more is controversial.^5^ The argument that Candida is not a pathogen in ventilator-associated pneumonia has been strengthened by a recent prospective study,^41^ although the diagnosis of infection was partly based on endotracheal aspirate cultures. Our decision to consider the presence of such organisms at 10^4^ CFU/mL or more in bronchoalveolar lavage fluid as ventilator-associated pneumonia was based on our previous studies and large clinical trials in suspected ventilator-associated pneumonia reporting substantial growth of these organisms.^34^, ^35^ However, we acknowledge that many clinicians would not consider fungi, yeasts, or Enterococci as pathogens in ventilator-associated pneumonia.

Controversy also surrounds the issue of whether patients with suspected ventilator-associated pneumonia should be included in trials if antibiotics have been started or adjusted in the 3 days before microbiological sampling, because of the theoretical risk of sterilising samples taken for culture.^5^ We elected to include such patients on the grounds that our derivation and validation studies had similar ventilator-associated pneumonia rates (24% and 35%, respectively), with the derivation study excluding and the validation study including such patients. The similar ventilator-associated pneumonia rate in the current study (34%) provides evidence that inclusion of such patients did not materially alter ventilator-associated pneumonia rates. However, although there was only one false negative result identified for our test, it remains theoretically possible that bronchoalveolar lavage fluid samples might be sterile for technical reasons, such as inadequate sampling or delays in analysis, as has been described for blood cultures.^42^, ^43^ We attempted to mitigate this risk by implementing a protocolised bronchoalveolar lavage, ensuring timely delivery of samples and having quality control checks within the biomarker assay.

Finally, landmark studies of blood biomarkers of infection have taken advantage of changes in diagnostic parameters over serial timepoints.^16^, ^19^, ^44^ This study was confined to a single timepoint based on our previous data, the desire to provide results well before bronchoalveolar lavage fluid culture results were available to clinicians, and because the mild inflammation associated with bronchoalveolar lavage would confound results from subsequent lavages. However, we cannot be certain that we used the optimum timepoint(s) for sampling.

In conclusion, biomarker-guided exclusion of ventilator-associated pneumonia did not reduce antibiotic use in centres that had committed to following test recommendations. Process evaluation suggested that lack of adoption of the technology and clinician behaviour had a greater influence on trial outcomes than did test performance. Antibiotic prescribing behaviours appear entrenched and recalcitrant to change. Future trials of diagnostic tests for ventilator-associated pneumonia should incorporate detailed implementation strategies informed by prior characterisation of factors that influence prescribing and diagnostic decision making.

## Data sharing

Data collected for this study will be made available (in the form of any or all from the de-identified data on the study database, study protocol, statistical analysis plan, and analytic code) to researchers who provide a methodologically sound research proposal, to assist with achievement of aims in the approved proposal. Data will be available from the time of publication of the Article in print. Proposals should be directed to the Newcastle Clinical Trials Unit.
