# Supplementary material for: Biomarker-guided antibiotic stewardship in suspected ventilator-associated pneumonia (VAPrapid2): a randomised controlled trial and process evaluation
Source: Lancet Respir Med. 2020 Feb;8(2):182–91. doi: 10.1016/S2213-2600(19)30367-4 (PMC7599318; doi:10.1016/S2213-2600(19)30367-4)
Supplement: Supplementary appendix [file mmc1.pdf]

THE LANCET
Respiratory Medicine

Supplementary appendix

This appendix formed part of the original submission and has been peer reviewed. We post it as supplied by the authors.

Supplement to: Hellyer TP, McAuley DF, Walsh TS, et al. Biomarker-guided antibiotic stewardship in suspected ventilator-associated pneumonia (VAPrapid2): a randomised controlled trial and process evaluation. *Lancet Respir Med* 2019; published online Dec 3. https://doi.org/10.1016/S2213-2600(19)30367-4.

VAPrapid-2 supplementary material

Contents

Sites and investigators 2

Data monitoring and safety committee 3

METHODS 4

Process evaluation 4

Statistical analysis 4

Definitions of level 2 and 3 care 5

Biomarker testing 5

RESULTS 6

Diagnostic performance of the assay 6

Graphical representation of the primary outcome 7

Antibiotic use in patients with VAP and non-VAP by trial arm 7

Microbiology 8

Antibiotic-resistant infections 8

Sensitivity analysis for the primary outcome 9

Sub-group analysis 10

Sub-group: Clinician assessment of likelihood of VAP 12

Sub-group: Medical vs Surgical 14

Sub-group: Medical vs Surgical (head injury/trauma) vs Surgical (non-head injury/trauma) 16

Indications for antibiotics 18

Adverse events 20

Safety parameters for BAL 21

Process evaluation 22

Health resource utilisation 24

Sites and investigators (* indicates Principal Investigator)

Belfast Health and Social Care Trust (Belfast City Hospital and Royal Victoria Hospital), Belfast: Ronan McMullan*, Danny McAuley, Griania White, Laura Creighton, Lia McNamee, Pauline McElhill, Vanessa Quinn, Leona Bannon, Paula Turtle, Jonathan Stirling, Mark Connolly, Niall Brown.

Chelsea & Westminster Hospital NHS Foundation Trust, London: Suveer Singh*, William Kelleher, Kylie Norrie, Sophie Holmes, Fiona Clark, Jamie Carungcong, Charlotte Graham, Louise Greathead, Rabiya Zia, John Porter.

City Hospitals Sunderland NHS Foundation Trust (Sunderland Royal Hospital), Sunderland: Alistair Roy*, Ashley Allan, Adam Bell, Julie Sheriff.

Countess of Chester Hospital NHS Foundation Trust, Chester: Nicole Robin*, Maria Faulkner, Helen Robertson.

Gateshead Health NHS Foundation Trust (Queen Elizabeth Hospital), Gateshead: Vanessa Linnett*, Jenny Ritzema.

Lancashire Teaching Hospitals NHS Foundation Trust (Preston Royal Hospital), Preston: Craig Spencer*, Alexandra Williams, Terrie-Louise Cromie, Jacqueline Baldwin.

Manchester University NHS Foundation Trust (Manchester Royal Infirmary), Manchester: Jonathan Bannard-Smith*, Anne Worthington, Elaine Coughlan, Katherine Wylie, Richard Clark, Andrew Brown, Rachel Pearson.

NHS Lothian (Royal Infirmary of Edinburgh and Western General Hospital), Edinburgh: Kallirroi Kefala (Royal Infirmary)*, Ross Paterson (Western)*, David Hope, Gosha Wojcik, Heidi Dawson, Corriene McCulloch, Martin McHugh, Alison Hardie.

Northumbria Healthcare NHS Foundation Trust (North Tyneside General Hospital, Wansbeck General Hospital, Northumbria Specialist Emergency Care Hospital), Northumbria: Bryan Yates*, Helen Bailey, Maureen Armstrong, Kirsten Walker.

Salford Royal NHS Foundation Trust (Salford Royal Hospital), Salford: Paul Dark*, Joy Dearden, Wendy Osbourne, Victoria O'Loughlin, Tracey Evans, Kathleen Leonard-Bedwell, Anne Worthington, Dionne Ellison, Samantha Fish, Kate Whatmough, Gayatri Chochan, .

Sandwell and West Birmingham Hospitals NHS Trust (City Hospital and Sandwell Hospital), Birmingham: Jonathan Hulme*, Brian Gammon, Fran Lloyd, Julie Colley.

South Tees Hospitals NHS Foundation Trust (James Cook University Hospital), Middlesbrough: Stephen Bonner*, Keith Hugill.

The Dudley Group NHS Foundation Trust (Russells Hall Hospital), Dudley: Julian Sonksen*, Sarah Fullwood, Ranjit Gidda, Sue Merotra, Clare Allcock.

The Newcastle upon Tyne Hospitals NHS Foundation Trust (Freeman Hospital and Royal Victoria Infirmary), Newcastle upon Tyne: Stephen Wright (Freeman)*, Simon Baudouin (RVI)*, Christine Boyd, Carmen Scott, Verity Calder, Craig Samson, Charley Higham, Audrey Perry, Kathryn Day, Daniel Tierney.

The Royal Liverpool and Broadgreen University Hospitals (Royal Liverpool University Hospital), Liverpool: Ingeborg Welters*, Anna Walker, Karen Williams, David Shaw, Samantha Hendry, Julie Patrick-Heselton, Victoria Waugh, Carol Paisley, James Watmore, Ben Morton.

University Hospitals Birmingham NHS Foundation Trust (Heartlands Hospital), Birmingham: Gavin Perkins*, Savita Gossain, Joanne Gresty, Keith Couper, Peter Sutton, Teresa Melody, Priti Rathod, Thomas Bodah, Samuel Green, Paul Connor, Kerstin Boese.

University Hospitals Coventry and Warwickshire (University Hospital Coventry), Coventry: Christopher Bassford*, Geraldine Ward, Jeffery Ting.

Newcastle Clinical Trials Unit: Jennie Parker, Jaki Begum, Cath Brennand

Data monitoring and safety committee

Prof Nigel Webster (Chair), Institute of Medical Sciences, University of Aberdeen

Prof Anthony Gordon, Faculty of Medicine, Department of Surgery and Cancer, Imperial College London

Prof Simon Gates, Warwick Clinical Trials Unit, University of Warwick

Prof Mervyn Singer, Faculty of Medicine, University College London

METHODS

Process evaluation

Process evaluation was carried out in three phases, with the first two informing the design of the third.

Pre-trial data were obtained from twenty-two ICUs. Data were collected by telephone interview with the site principal investigators (PIs) for the first 14 ICUs participating in the trial. In the case of seven ICUs joining the study after the first fourteen, PIs were interviewed during site initiation visits. In the case of the final site, a telephone interview was conducted with the PI. Interviews explored pre-trial practice in the clinical diagnosis of VAP, management of VAP and the influence of laboratory tests, and current use of BAL.

Within-trial data were collected after the trial had been open to recruitment for at least one year at individual ICUs. Research nurses at 22 ICUs were interviewed by telephone. Themes systematically analysed included quality of intervention delivery, identification of facilitators and barriers to compliance with different elements of the intervention, and delineation of internal and external factors that might influence implementation. Content analysis was undertaken using notes generated at sites.

Data accumulated in 22 ICUs studied in the pre-trial and within-trial elements were used to categorise units on two levels, i.e. level of perceived risk of the intervention (high or low), and (b) reported experience of the trial (positive or negative). Units therefore fell into one of 4 categories (high perceived risk, negative perception; high perceived risk, positive perception, low perceived risk, negative perception; and low perceived risk, positive perception). A purposive set of 9 ICUs was determined, with all 4 categories represented, capturing the maximum variation of characteristics. The late-trial analysis included these 9 ICUs, with interviews conducted in the last three months of the intervention period. At each ICU, at least one research nurse, PI and ward manager were interviewed, though additional doctors and research nurses could be interviewed if they had trial responsibilities. Four of the sites were hubs for biomarker testing, and at each of these a laboratory technician was also interviewed.

Late-trial interviews sought to examine: the fidelity of the intervention; factors influencing conversion from eligibility to recruitment; ways in which the specific context of each unit influenced implementation of the trial; procedures used to recruit patients into the trial; and attitudinal factors influencing delivery and implementation. Interviews were recorded digitally and transcribed verbatim. Data were analysed thematically with a framework approach. Data from individual interviewees were pooled to yield nine unit-level summaries. These were assessed alongside quantitative trial data. In accordance with recruitment rates derived from the within-trial sample, late trial analysis considered ICUs in two groups, namely high recruiters ($\geq$ 50% conversion of eligibility to recruitment) and low recruiters (< 50% conversion).

Throughout the trial, when a biomarker-guided recommendation to discontinue antibiotics was issued and where there was non-compliance with the recommendation, PIs were asked to complete a questionnaire assessing the reason(s), and their confidence in the biomarker result (on a scale of 1-4). The process evaluation incorporated assessment of antibiotic prescribing and the indications given, and these data were scrutinised in cases where no questionnaire was completed.

Statistical analysis

The discrete-time Cox model (analysis A) and the approach of re-setting AFDs to zero for any patient that died in the 7-day observation period (analysis B) are presented in the results below. For the purpose of the sensitivity analysis, the laboratory testing site was the ‘centre’ as patient recruitment in individual ICUs was in some cases too low to use ICU as the ‘centre’. For sub-group analyses, the approach taken in Analysis A, was that if the p value was significant, separate models were fitted within each subgroup level; if not significant, the additive effect of subgroups was reported via relevant HR and 95% CI.

Secondary outcomes (antibiotic-days at days 7, 14 and 28; AFD at days 14 and 28; duration of critical care stay; duration of hospital stay; and lengths of level 2 and level 3 stay) were analysed using Cox proportional hazards models. 28-day mortality, critical care mortality and presence of antibiotic-associated infections were analysed by logistic regression. SOFA score at days 3, 7 and 14 was analysed by linear regression. Ventilator-free days (VFD) at day 28 and the number of antibiotic-resistant pathogens were analysed by Poisson regression.

Health resource use was summarised by descriptive statistics for each group and non-parametric bootstrapping used to calculate 95% bootstrap confidence interval. Multiple regression analysis was used to identify characteristics associated with cost.

Definitions of level 2 and 3 care

Level 2 care refers to high dependency unit care, i.e. patients in receipt of single organ support or basic respiratory support, or in step-down from level 3 (ICU).

Level 3 care refers to intensive care unit care, i.e. patients in receipt of advanced respiratory support or support of 2 or more organs.

Biomarker testing

Samples were transported for biomarker testing to one of six testing hubs. These were in Newcastle upon Tyne, Edinburgh, Belfast, Salford, Birmingham and London (Chelsea & Westminster,) with the aim of all samples being able to reach a hub within 1.5 hours.

RESULTS

Diagnostic performance of the assay

Statistic	Value	95% CI
Sensitivity	96·8%	83·3% - 99·9%
Specificity	34·2 %	19·6% - 51·4%
Positive Likelihood Ratio	1·47	1·16 – 1·87
Negative Likelihood Ratio	0·09	0·01 – 0·68
Positive Predictive Value	54·6%	48·6% - 60·4%
Negative Predictive Value	92·9 %	64·3% - 99·0%

Table S1: Diagnostic performance of the biomarker assay to diagnose VAP.
Confirmed VAP was defined as culture of a potentially pathogenic organism $\geq 10^4$ colony forming units/ml. Diagnostic parameters calculated from patients with available microbiology and biomarker data (n=69).

Graphical representation of the primary outcome (antibiotic-free days in the 7 days following BAL)

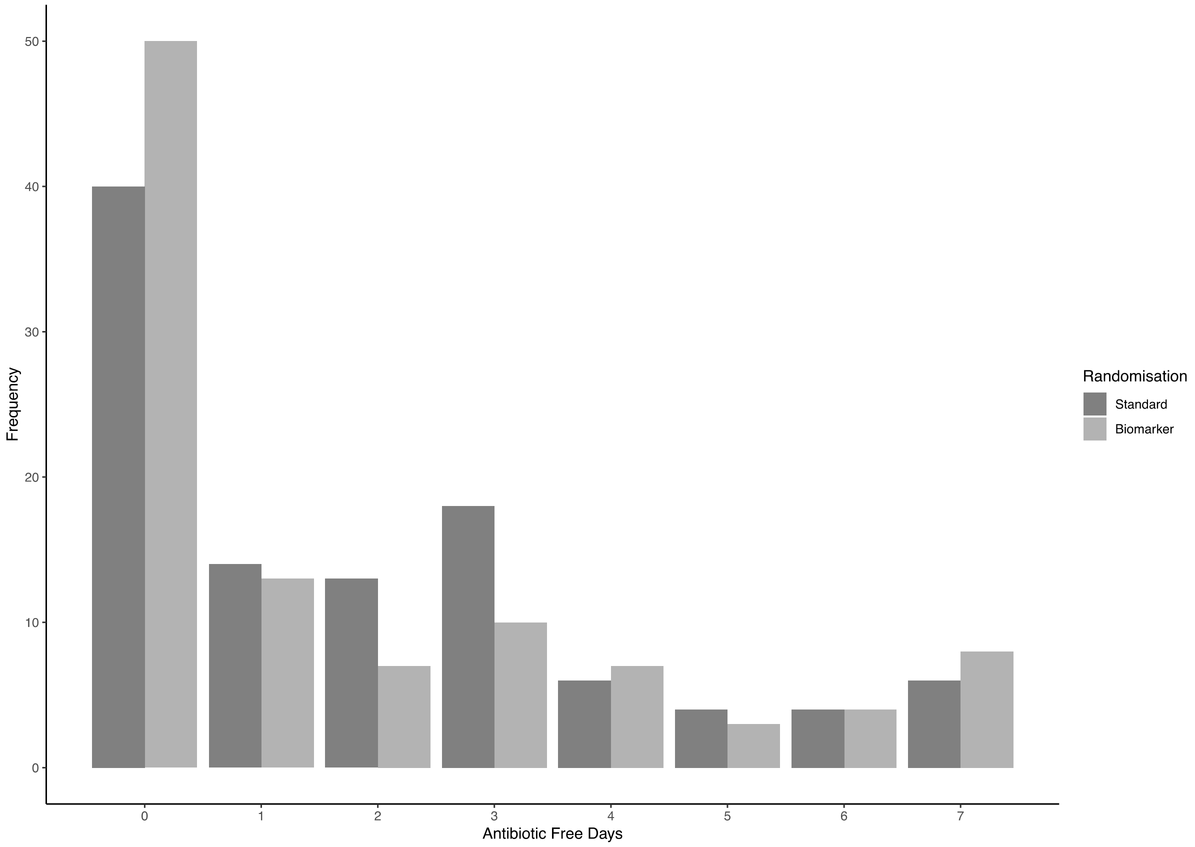

Figure S1: Distribution of antibiotic-free days at day 7.

Antibiotic use in patients with VAP and non-VAP by trial arm

	**Biomarker-guided recommendation on antibiotics**			**Routine use of antibiotics**		
	**N**	**Median**	**Interquartile range**	**N**	**Median**	**Interquartile range**
**Confirmed VAP**	38	0·0	0·0-2·0	32	0·5	0·0-3·0
**No confirmation of VAP**	64	1·0	0·0-3·2	73	2·0	0·0-3·0

Table S2: Antibiotic-free days to day 7 in patients with and without confirmed VAP.

Microbiology

**Organisms grown from BAL fluid**	**AFD at day 7**
Sterile	0
Candida spp.	0
Enterobacter cloacae<br>*Haemophilus influenzae*	0
Sterile	0
Enterococcus spp.*	1
Klebsiella pneumoniae<br>*Pseudomonas aeruginosa*	1
Sterile	2
Sterile	2
Sterile	3
Enterobacter cloacae<br>*Citrobacter braakii*<br>*Klebsiella oxytoca*<br>*Fusarium* spp.	3
Sterile	4
Proteus mirabilis	6
Sterile	6
Sterile	7
Sterile	7
Sterile	7
Sterile	7

Table S3: Characteristics of the seventeen patients with a low IL-1β/IL-8 test result.

Patients with 7 AFD are those for whom antibiotics were considered to have been discontinued appropriately, i.e. the discontinuation recommendation was followed. *=organism cultured at $\geq 10^4$ CFU/mL, i.e. the patient had VAP; all other organisms listed were cultured at $<10^4$ CFU/mL.

Antibiotic-resistant infections

There were 6 new antibiotic-associated infections recorded in the intervention arm and 7 in the control arm. The number of antibiotic-resistant pathogens appeared to suggest a significantly increased risk in the intervention arm, though this was strongly influenced by a single outlier with multiple bacterial pathogens. The difference was not observed when the outlier was removed.

Sensitivity analysis for the primary outcome

The following analyses (A&B) were carried out within the intention-to-treat (ITT) population.

A. Discrete-time Cox model: This uses discrete time survival analysis via the discSurv package in R to model AFDs at 7 days by fitting a parameter for the hazard ratio in the randomised study arm relative to the standard treatment arm and adjusting for centre effects. Thus, any patients dying within 7 days contribute some AFD information, but are censored at that point. The hazard ratio is for the occurrence of antibiotic use and a HR <1 corresponds to decreased hazard of the start of antibiotic use in the intervention arm relative to the control arm.

B. The primary analysis (chi-squared test from 2x8 cross-tabulation of arm by AFD) was repeated, but with AFD set equal to zero for any patients dying within the initial 7-day monitoring period.

Sensitivity analysis A:

**HR**	**95% CI**
1·08	(0·75, 1·56)

Table S4: Discrete-time Cox sensitivity analysis for primary outcome in the ITT population.

Sensitivity analysis B:

	**AFD (deaths re-set to 0)**								
**Arm**	**0**	**1**	**2**	**3**	**4**	**5**	**6**	**7**	**Total**
Routine use of antibiotics (n)	51	13	13	16	3	1	3	5	105
	48·5%	12·4%	12·4%	15·2%	2·9%	1·0%	2·9%	4·8%	
Biomarker-guided recommendation on antibiotics (n)	60	12	6	7	6	2	3	6	102
	58·8%	11·8%	5·9%	6·9%	5·9%	2·0%	2·9%	5·9%	

Table S5: Sensitivity analysis for the primary outcome in the ITT population with AFD set to zero for patients who died in the 7-day period. Chi-squared test: p=0·31 (7 df, chi-squared = 8·25), Fisher's Exact test: p = 0·30.

Sub-group analysis

In subgroup analyses, there were no significant differences in primary and secondary outcome measures in the per-protocol analyses. When stratifying by clinician assessment of likelihood of VAP fewer AFDs were observed in the 'high' likelihood group of the intervention arm (Fisher's exact test p=0·033). In the discrete-time Cox proportional hazard model, trial arm did not significantly influence the model (p=0·98), although the model did demonstrate more antibiotic use in the 'high' likelihood group ('high' vs 'low': HR 2·36 [95% CI 1·21-4·91]). There were no differences between trial arms when analysing the strata of 'medical', 'surgical', or 'head injury/trauma'.

Per-protocol sensitivity analysis:

Sensitivity analysis A&B, as described above.

Sensitivity analysis A:

**HR**	**95% CI**
1·26	(0·85, 1·87)

Table S6: Per-protocol discrete-time Cox sensitivity analysis.

Sensitivity analysis B:

	**AFD (deaths re-set to 0)**								
**Arm**	**0**	**1**	**2**	**3**	**4**	**5**	**6**	**7**	**Total**
Routine use of antibiotics (n)	51	13	13	16	3	1	3	5	105
	48·5%	12·4%	12·4%	15·2%	2·9%	1·0%	2·9%	4·8%	
Biomarker-guided recommendation on antibiotics (n)	48	10	6	4	5	1	2	4	80
	60·0%	12·5%	7·5%	5·0%	6·2%	1·2%	2·5%	5·0%	

Table S7: Sensitivity analysis for the primary outcome in the per-protocol population with AFD set to zero for patients who died in the 7-day period.

Chi-squared test: p=0·35 (7 df, chi-sq statistic = 7·84). Fisher's Exact test: p = 0·30.

Per-protocol secondary outcome measures

Outcome	Sample Size	Number of Events	HR	95% CI
Antibiotic Days (7 Days post-BAL)	185	167	0·80	(0·59, 1·10)
Antibiotic Days (14 Days post-BAL)	170	149	0·90	(0·64, 1·25)
Antibiotic Days (28 Days post-BAL)	158	133	0·94	(0·66, 1·33)
AFD (14 Days post-BAL)	170	149	1·17	(0·84, 1·63)
AFD (28 Days post-BAL)	158	133	1·03	(0·73, 1·46)
Duration critical care stay	182	145	1·06	(0·75, 1·50)
Duration hospital stay	183	144	0·86	(0·61, 1·22)
Length level 3 stay	182	145	1·07	(0·76, 1·51)
Length level 2 stay	133	130	1·11	(0·77, 1·60)

Table S8: Per-protocol secondary outcome measures.
Data were analysed by Cox proportional hazards and are presented as hazard ratio (HR).

Outcome	Sample Size	OR	95% CI
Mortality at 28 days	186	1·26	(0·60, 2·64)
Critical care mortality	186	1·25	(0·59, 2·66)
Presence of antibiotic-associated infections to 56 days	185	0·86	(0·24, 2·87)

Table S9: Per-protocol secondary outcome measures.
Data were analysed by logistic regression and are presented as odds ratio (OR).

Outcome	Sample Size	Mean Diff.	95% CI
SOFA at 3 days	174	-0·18	(-0·94, 0·59)
SOFA at 7 days	178	-0·14	(-1·17, 0·36)
SOFA at 14 days	178	-0·28	(-1·09, 0·53)

Table S10: Per-protocol secondary outcome measures.
Data were analysed by linear regression and are presented as mean difference.

Outcome	Sample Size	Risk Ratio	95% CI
VFD (at 28 days)	186	1·11	(1·02, 1·21)
Number of antibiotic-resistant pathogens (at 56 days)	185	1.·85	(1·24, 2·79)
Number of pathogens (outlier excluded)	184	1·46	(0·95, 2·25)

Table S11: Per-protocol secondary outcome measures.
Data were analysed by Poisson regression and are presented as risk ratio.

Sub-group: Clinician assessment of likelihood of VAP

The primary analysis was not model-based and so a sub-group covariate could not be fitted and tested for an interaction. Therefore, the analysis was stratified.

Clinical assessment of VAP likelihood = Low

	AFD								
**Arm**	**0**	**1**	**2**	**3**	**4**	**5**	**6**	**7**	**Total**
Routine use of antibiotics (n)	2	1	0	2	1	0	0	1	7
	28·6%	14·3%	0·0%	28·6%	14·3%	0·0%	0·0%	14·3%	
Biomarker-guided recommendation on antibiotics (n)	1	0	2	1	2	0	2	1	9
	11·1%	0·0%	22·2%	11·1%	22·2%	0·0%	22·2%	11·1%	

Table S12: Sub-group assessment where the clinician assessed the likelihood of VAP to be low.
Chi-squared test: p=0·44 (6 df, chi-sq statistic = 5·84); Fisher's Exact test: p = 0·67.

Clinical assessment of VAP likelihood = Medium

	AFD								
**Arm**	**0**	**1**	**2**	**3**	**4**	**5**	**6**	**7**	**Total**
Routine use of antibiotics (n)	20	8	4	7	2	3	4	3	51
	39·2%	15·7%	7·8%	13·7%	3·9%	5·9%	7·8%	5·9%	
Biomarker-guided recommendation on antibiotics (n)	14	5	3	3	1	2	1	4	33
	42·4%	15·2%	9·1%	9·1%	3·0%	6·1%	3·0%	12·1%	

Table S13: Sub-group clinician assessment where the clinician assessed the likelihood of VAP to be medium.
Chi-squared test: p=0·95 (7 df, chi-sq statistic = 2·22); Fisher's Exact test: p = 0·96.

Clinical assessment of VAP likelihood = High

	AFD								
**Arm**	**0**	**1**	**2**	**3**	**4**	**5**	**6**	**7**	**Total**
Routine use of antibiotics (n)	18	5	9	9	3	1	0	2	47
	38·3%	10·6%	19·1%	19·1%	6·4%	2·1%	0·0%	4·3%	
Biomarker-guided recommendation on antibiotics (n)	35	8	1	6	4	1	1	3	59
	59·3%	13·6%	1·7%	10·2%	6·8%	1·7%	1·7%	5·1%	

Table S14: Sub-group clinician assessment where the clinician assessed the likelihood of VAP to be high.
Chi-squared test: p=0·07 (7 df, chi-sq statistic = 13·30); Fisher's Exact test: p = 0·033.

Sensitivity analysis:

The following two sensitivity analysis procedures were carried out in remaining sub-group analyses:
A: Discrete-time Cox model. The Statistical Analysis Plan stated that if the p value was significant, the next step would be to fit separate models within each subgroup level; if not significant, the plan was to quote the additive effect of subgroups via relevant HR and 95% CI. HR<1 corresponds to decreased hazard of the start of antibiotic use in the appropriate subgrouping relative to its baseline.
B. Replacement of AFD by zero for patients who died during the 7-day observation period.

Sensitivity analysis A:

**Subgroup**	**p-value for Interaction with Arm**	**HR for Antibiotic Use in Subgroups (additive)**	**95% CI**
Likelihood of VAP	0·98	Medium/ Low: 1·88<br>High/ Low: 2·36	(0·95, 3·95)<br>(1·21, 4·91)

Table S15: Sensitivity analysis - clinician assessment of likelihood of VAP.
Data were analysed using a discrete-time Cox model.

Sensitivity analysis B:

	**AFD**								
**Arm**	**0**	**1**	**2**	**3**	**4**	**5**	**6**	**7**	**Total**
Routine use of antibiotics (n)	4	1	0	2	0	0	0	0	7
	57·1%	14·3%	0·0%	28·6%	0·0%	0·0%	0·0%	0·0%	
Biomarker-guided recommendation on antibiotics (n)	2	0	2	1	2	0	1	1	9
	22·2%	0·0%	22·2%	11·1%	22·2%	0·0%	11·1%	11·1%	

Table S16: Sensitivity analysis, where the clinician assessed the likelihood of VAP to be low. Replacement of the AFD by zero for patients who died. Chi-squared test: p=0·25 (6 df, chi-sq statistic = 7·87); Fisher's Exact test: p = 0·34.

	**AFD**								
**Arm**	**0**	**1**	**2**	**3**	**4**	**5**	**6**	**7**	**Total**
Routine use of antibiotics (n)	26	7	4	6	1	1	3	3	51
	51·0%	13·7%	7·8%	11·8%	2·0%	2·0%	5·9%	5·9%	
Biomarker-guided recommendation on antibiotics (n)	17	5	3	2	1	1	1	3	33
	51·5%	15·2%	9·1%	6·1%	3·0%	3·0%	3·0%	9·1%	

Table S17: Sensitivity analysis, where the clinician assessed the likelihood of VAP to be medium.
Replacement of the AFD by zero for patients who died. Chi-squared test: p=0·98 (7 df, chi-sq statistic = 1·58); Fisher's Exact test: p = 0·98.

	AFD								
**Arm**	**0**	**1**	**2**	**3**	**4**	**5**	**6**	**7**	**Total**
Routine use of antibiotics (n)	21	5	9	8	2	0	0	2	47
	44·7%	10·6%	19·1%	17·0%	4·3%	0·0%	0·0%	4·3%	
Biomarker-guided recommendation on antibiotics (n)	41	7	0	4	3	1	1	2	59
	69·5%	11·9%	0·0%	6·8%	5·1%	1·7%	1·7%	3·4%	

Table S18: Sensitivity analysis, where the clinician assessed the likelihood of VAP to be high. Replacement of the AFD by zero for patients who died. Chi-squared test: p=0·011 (7 df, chi-sq statistic = 18·19); Fisher's Exact test: p = 0·002.

Sub-group: Medical vs Surgical

	AFD								
**Arm**	**0**	**1**	**2**	**3**	**4**	**5**	**6**	**7**	**Total**
Routine use of antibiotics (n)	23	12	9	12	6	2	3	2	69
	33·3%	17·4%	13·0%	17·4%	8·7%	2·9%	4·3%	2·9%	
Biomarker-guide recommendation on antibiotics (n)	26	8	4	8	4	1	2	6	59
	44·1 %	13·6%	6·8%	13·6%	6·8%	1·7%	3·4%	10·2%	

Table S19: Sub-group analysis for the medical admission category.
Chi-squared test: p=0·55 (7 df, chi-sq statistic = 5·90); Fisher's Exact test: p = 0·57.

	AFD								
**Arm**	**0**	**1**	**2**	**3**	**4**	**5**	**6**	**7**	**Total**
Routine use of antibiotics (n)	17	2	4	6	0	2	1	4	36
	47·2%	5·6%	11·1%	16·7%	0·0%	5·6%	2·8%	11·1%	
Biomarker-guided recommendation on antibiotics (n)	24	5	3	2	3	2	2	2	43
	55·8%	11·6%	7·0%	4·7%	7·0%	4·7%	4·7%	4·7%	

Table S20: Sub-group analysis for the surgical admission category. Chi-squared test: p=0·33 (7 df, chi-sq statistic = 8·07); Fisher's Exact test: p = 0·36.

Sensitivity analysis A:

Subgroup	p-value for Interaction with Arm	HR for Antibiotic Use in Subgroups (additive)	95% CI
Admission Category (M/S)	0·36	Surg/ Med: 1·15	(0·78, 1·69)

Table S21: Sensitivity analysis, Medical versus Surgical.
Data were analysed using a discrete-time Cox model.

Sensitivity analysis B:

	**AFD**								
**Arm**	**0**	**1**	**2**	**3**	**4**	**5**	**6**	**7**	**Total**
Routine use of antibiotics (n)	32	11	9	10	3	0	2	2	69
	46·4%	15·9%	13·0%	14·5%	4·3%	0·0%	2·9%	2·9%	
Biomarker-guided recommendation on antibiotics (n)	33	7	3	6	4	1	1	4	59
	55·9%	11·9%	5·1%	10·2%	6·8%	1·7%	1·7%	6·8%	

Table S22: Sensitivity analysis for the medical admission category.
AFDs were replaced by zero for patients who died. Chi-squared test: p=0·51 (7 df, chi-sq statistic = 6·30); Fisher’s Exact test: p = 0·52**.**

	**AFD**								
**Arm**	**0**	**1**	**2**	**3**	**4**	**5**	**6**	**7**	**Total**
Routine use of antibiotics (n)	19	2	4	6	0	1	1	3	36
	52·8%	5·6%	11·1%	16·7%	0·0%	2·8%	2·8%	8·3%	
Biomarker-guided recommendation on antibiotics (n)	27	5	3	1	2	1	2	2	43
	62·8%	11·6%	7·0%	2·3%	4·7%	2·3%	4·7%	4·7%	

Table S23: Sensitivity analysis for the surgical admission category.
AFDs were replaced by zero for patients who died. Chi-squared test: p=0·30 (7 df, chi-sq statistic = 8·37); Fisher’s Exact test: p = 0·29.

Sub-group: Medical vs Surgical (head injury/trauma) vs Surgical (non-head injury/trauma)

For the medical admission category, the data are as represented in Table S25.

	AFD								
**Arm**	**0**	**1**	**2**	**3**	**4**	**5**	**6**	**7**	**Total**
Routine use of antibiotics (n)	6	1	1	2	0	1	1	3	15
	40·0%	6·7%	6·7%	13·3%	0·0%	6·7%	6·7%	20·0%	
Biomarker-guided recommendation on antibiotics (n)	10	0	2	1	2	1	0	1	17
	58·8%	0·0%	11·8%	5·9%	11·8%	5·9%	0·0%	5·9%	

Table S24: Sub-group analysis for the surgical admission (head injury or trauma) category. Chi-squared test: p=0·48 (7 df, chi-sq statistic = 6·57); Fisher's Exact test: p = 0·57.

	AFD								
**Arm**	**0**	**1**	**2**	**3**	**4**	**5**	**6**	**7**	**Total**
Routine use of antibiotics (n)	11	1	3	4	0	1	0	1	21
	52·4%	4·8%	14·3%	19·0%	0·0%	4·8%	0·0%	4·8%	
Biomarker-guided recommendation on antibiotics (n)	14	5	1	1	1	1	2	1	26
	53·8%	19·2%	3·8%	3·8%	3·8%	3·8%	7·7%	3·8%	

Table S25: Sub-group analysis for the surgical admission (non-head injury or trauma) category. Chi-squared test: p=0·30 (7 df, chi-sq statistic = 8·39); Fisher's Exact test: p = 0·28.

Sensitivity analysis A:

**Subgroup**	**p-value for Interaction with Arm**	**HR for Antibiotic Use in Subgroups (additive)**	**95% CI**
Admission Category M/ S(head)/ S (other)	0·40	S (Head)/ M: 0·79<br>S (Other)/ M: 1·52	(0·46, 1·34)<br>(0·95, 2·43)

Table S26: Sensitivity analysis, medical vs surgical (head injury/trauma) vs surgical (non-head injury/trauma). Discrete-time Cox model.

Sensitivity analysis B:
For the medical category the data are as presented in Table S25.

	AFD								
**Arm**	**0**	**1**	**2**	**3**	**4**	**5**	**6**	**7**	**Total**
Routine use of antibiotics (n)	8	1	1	2	0	0	1	2	15
	53·3%	6·7%	6·7%	13·3%	0·0%	0.0%	6·7%	13·3%	
Biomarker-guided recommendation on antibiotics (n)	12	0	2	0	1	1	0	1	17
	70·6%	0·0%	11·8%	0·0%	5·9%	5·9%	0·0%	5·9%	

Table S27: Sensitivity analysis for the surgical admission (head injury or trauma) category.
AFDs were replaced by zero for patients who died. Chi-squared test: p=0·39 (7 df, chi-sq statistic = 7·37); Fisher's Exact test: p = 0·50.

	AFD								
**Arm**	**0**	**1**	**2**	**3**	**4**	**5**	**6**	**7**	**Total**
Routine use of antibiotics (n)	11	1	3	4	0	1	0	1	21
	52·4%	4·8%	14·3%	19·0%	0·0%	4·8%	0·0%	4·8%	
Biomarker-guided recommendation on antibiotics (n)	15	5	1	1	1	0	2	1	26
	57·7%	19·2%	3·8%	3·8%	3·8%	0·0%	7·7%	3·8%	

Table S28: Sensitivity analysis for the surgical admission (non-head injury or trauma) category.
AFDs were replaced by zero for patients who died. Chi-squared test: p=0·21 (7 df, chi-sq statistic = 9·66); Fisher's Exact test: p = 0·17.

Indications for antibiotics

	Biomarker-guided recommendation on antibiotics		Routine use of antibiotics	
**Indication**	**N**	**%**	**N**	**%**
Community-acquired pneumonia	8	9·6	6	6·9
Hospital-acquired pneumonia	9	10·8	16	18·4
Ventilator-associated pneumonia	33	39·8	35	40·2
Intra-abdominal infection	7	8·4	3	3·4
Urinary infection	0	0·0	1	1·1
Skin infection	2	2·4	3	3·4
Central nervous system infection	1	1·2	0	0·0
Prophylaxis (incl. topical antibiotics)	4	4·8	4	4·6
Other	19	22·9	19	21·8
**Totals**	83		87	

Table S29: Stated antibiotic indications at baseline.

	Biomarker-guided recommendation on antibiotics		Routine use of antibiotics	
**Indication**	**N**	**%**	**N**	**%**
Community-acquired pneumonia	0	0·0	4	5·1
Hospital-acquired pneumonia	7	9·9	11	14·1
Ventilator-associated pneumonia	45	63·4	32	41·0
Intra-abdominal infection	2	2·8	2	2·6
Urinary infection	0	0·0	0	0·0
Skin infection	1	1·4	4	5·1
Central nervous system infection	2	2·8	2	2·6
Other	14	19·7	23	29·5
**Totals**	71		78	

Table S30: Stated antibiotic indications at day 7.

	Biomarker-guided recommendation on antibiotics		Routine use of antibiotics	
**Indication**	**N**	**%**	**N**	**%**
Community-acquired pneumonia	0	0·0	0	0·0
Hospital-acquired pneumonia	3	8·3	2	6·0
Ventilator-associated pneumonia	13	36·1	10	30·3
Intra-abdominal infection	0	0·0	1	3·0
Urinary infection	2	5·6	1	3·0
Skin infection	1	2·8	3	9·1
Central nervous system infection	2	5.6	3	9.1
Other	15	41·7	13	39·4
**Totals**	36		33	

Table S31: Stated antibiotic indications at day 14.

	Biomarker-guided recommendation on antibiotics		Routine use of antibiotics	
**Indication**	**N**	**%**	**N**	**%**
Community-acquired pneumonia	0	0·0	0	0·0
Hospital-acquired pneumonia	6	19·4	5	21·7
Ventilator-associated pneumonia	4	12·9	4	17·4
Intra-abdominal infection	1	3·2	1	4·3
Urinary infection	3	9·7	1	4·3
Skin infection	4	12·9	1	4·3
Central nervous system infection	1	3·2	0	0·0
Other	12	38·7	11	47·8
**Totals**	31		23	

Table S32: Stated antibiotic indications at day 28.

Adverse events

The total number of AEs are described in Table S2. A summary of serious adverse events is shown in Table S3 (for the intervention arm) and in Table S4 (for the control arm).

	Biomarker-guided recommendation on antibiotics		Routine use of antibiotics	
	Number	%	Number	%
**With either AE or SAE**	56	-	40	-
- AE recorded only	51	91·7	38	95·0
- SAE recorded	5	8·9	2	5·0
**AEs**				
Severity: Mild	37	72·5	29	76·3
Moderate	12	23·5	7	18·4
Severe	1	2·0	2	5·3
(Missing)	1		0	
Related to Treatment: Unrelated	5	9·8	6	15·8
Unlikely	3	5·9	4	10·5
Possible	12	23·5	7	18·4
Probable	21	41·2	12	31·6
Definitely	9	17·7	9	23·7
(Missing)	1		0	
**SAEs**				
Severity: Mild	0	0·0	0	0·0
Moderate	1	20·0	0	0·0
Severe	4	80·0	2	100·0
Related to Treatment: Unrelated	3	60·0	2	100·0
Unlikely	1	20·0	0	0·0
Possible	0	0·0	0	0·0
Probable	0	0·0	0	0·0
Definitely	1	20·0	0	0·0

Table S33: Adverse event number, seriousness and causality.

SAE No.	Description	Severity	Related to treatment
003	VF arrest	Severe	Unlikely
004	Pneumothorax	Severe	Unrelated
005	Patient died approximately 10 hrs after BAL	Severe	Unrelated
006	Endotracheal tube displaced	Moderate	Definitely
007	Sub-arachnoid haemorrhage	Severe	Unrelated

Table S14: Serious adverse event summary for the biomarker-guided recommendation on antibiotics arm. VF, ventricular fibrillation.

SAE No.	Description	Severity	Related to treatment
001	Gastrointestinal bleed. Death.	Severe	Unrelated
002	Tension pneumothorax, cardiac arrest. Death.	Severe	Unrelated

Table S35: Serious adverse event summary for the routine use of antibiotics arm.

Safety parameters for BAL

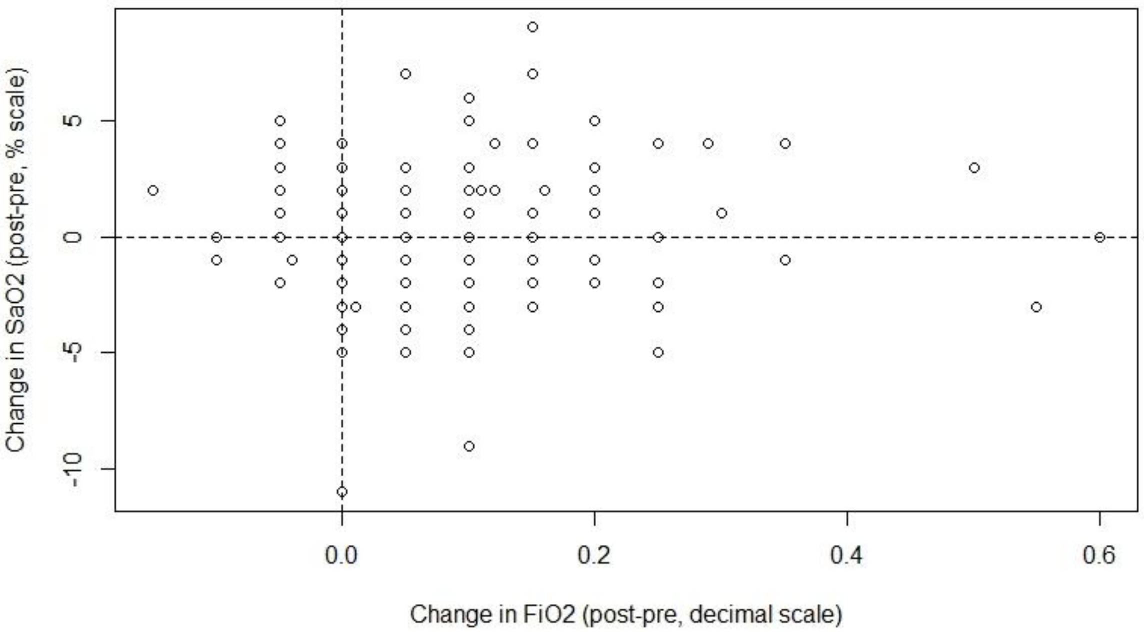

Figure S2: Change in oxygen saturations (SaO_2) and inspired oxygen fraction (FiO_2) in the 2 hours after BAL. Oxygen saturations and fraction of inspired oxygen were recorded before BAL (before pre-oxygenation) and 2 hours after bronchoscopy. The graph shows the change in pre- and post-BAL values. SaO_2 is expressed as a percentage. Five patients were excluded due to recording errors in FiO_2.

Process evaluation

Pre-trial analysis

Data were collected from 22 principal investigators (PIs, i.e. one PI from each of 22 ICUs). Use of bronchoscopy and BAL was described as relatively common practice in 4 of 22 units. In the remaining units, non-directed “mini-BAL” or endotracheal aspirates were more commonly used in the diagnosis of VAP. Commonly identified challenges in the diagnosis of VAP diagnosis included a lack of internationally accepted diagnostic criteria and absence of rapid response microbiology.

Factors associated with limited use of bronchoscopic BAL included: limited training and expertise among the pool of clinicians on the ICU; technical inexperience; and the potential barrier of difficult airway management. In addition, there was a preference for less invasive procedures on the grounds of being less technically complicated, requiring less resource and staff, and lower dependence on consultant-level involvement. PIs also cited a lack of evidence for added value from BAL over alternative diagnostic procedures. Finally, several PIs did not consider a negative culture from BAL as definitive proof of the absence of VAP.

Fourteen units routinely prescribed broad-spectrum antibiotic where there was an initial suspicion of VAP. Seven ICUs described a preference to hold off antibiotic treatment until culture results were returned when patients appeared stable, with a low threshold for empirical broad-spectrum antibiotics for deteriorating patients. The remaining ICU took a protocolised approach to management of suspected VAP, with the clinical pulmonary infection score (CPIS) triggering a chest x-ray, and antibiotics being commenced if there was evidence of new consolidation.

PIs were asked to consider their antibiotic prescribing in patients already on antibiotics, where a subsequent test result excluded VAP. Twelve PIs stated that usual practice within the ICU was to continue antibiotics for the duration of the prescribed course, regardless of test results. Six PIs stated that the decision would rest with the consultant in charge, with antibiotics only being stopped if there was accompanying clinical improvement. Four PIs described stopping antibiotics in accordance with negative test results/microbiology guidance.

All PIs alluded to difficulties in diagnosing site-specific infection in complex ICU patients, and said that stopping antibiotics was linked to the clinician’s perception of risk.

Within-trial analysis

Data were collected from 22 research nurses (i.e. one from each of 22 ICUs). Several units described that screening and recruitment were impaired by insufficient research nurse cover, which was often unpredictable. Only one research nurse perceived there was adequate cover for screening throughout the trial, reporting high recruitment of eligible patients. In general, research nurses with more than two years’ experience were more confident around recruitment, starting the process individually, and earlier.

Two barriers to laboratory sampling were identified. The first comprised a general requirement for the BAL sample to leave the ICU early in the working day to ensure timely processing (especially when transport of samples to a distant hub was required). As ICUs are generally at their busiest in the morning, there was a pressure on obtaining a timely BAL, compounded in some cases by competition for use of bronchoscopes shared with other clinical areas. The second related to inconsistent laboratory cover, owing to a limited pool of laboratory staff trained to process samples. Together, these factors were considered to have contributed to low recruitment. Despite these limitations, research nurses from 17 ICUs considered the trial to be relatively straightforward to deliver.

Perception of risk emerged as an important consideration influencing recruitment and delivery of the intervention. Research nurses in seven ICUs felt that the protocol’s requirement for a willingness to discontinue antibiotics if a biomarker test was negative led to some clinicians not recruiting patients, on the basis of being uncomfortable with the prospect of stopping antibiotics on the basis of the trial recommendation. Research nurses at the same sites perceived an apprehension around the use of bronchoscopic BAL as a diagnostic tool, with the volume of saline stipulated in the protocol adding to this feeling.

Attitudes toward the trial varied widely. Research nurses at five ICUs reported a lack of enthusiasm among clinicians, while the other 17 described generally positive/enthusiastic attitudes. Generally, doctors and nurses not actively involved in research were considered the most challenging to engage with the study. Other factors potentially impacting recruitment included waning enthusiasm over time in a trial with low eligibility and

infrequent recruitment on any given ICU, and potentially greater enthusiasm for other trials open on the same unit.

Late-trial analysis

Face-to-face interviews were completed with nine PIs, thirteen research nurses, nine ward managers, five doctors who shared PI duties and four laboratory technicians. The nine ICUs involved were sub-divided into two categories, based on recruitment levels observed at the time of interviews, i.e. high recruiters (≥ 50% conversion from eligibility to recruitment; n=2) and low recruiters (< 50% conversion; n=7).

Both high-recruiting units had dedicated ICU research nurses. Routine use of BAL in the diagnosis of suspected VAP was common, and both units had a strong culture of limiting the use of, and de-escalating, antibiotics. Antibiotics were only commenced upon culture confirmation of VAP, unless the patient was very unwell. Both units had trial champions (a PI or other senior clinician) who continuously ensured study awareness was high, and encouraged enthusiasm amongst the teams. The trial champions were available for patient recruitment most of the time, and sustained recruitment appeared to enhance visibility of the trial. The importance of trial champions was emphasised in one unit by a fall in recruitment:eligibility from 1.0 (within-trial analysis) to 0.56 (late-trial analysis), with the trial champion leaving the ICU shortly after the within-trial assessment.

In both high-recruiting units, the study workload was largely confined to dedicated research teams with little impact upon non-research staff working clinically within the units. Both units reported that once patients were recruited into the study, intervention delivery was efficient and processes easy to follow.

Among the seven low-recruiting units, three had dedicated ICU research nurses, but only one had worked clinically in an ICU, leading to perceived barriers to integration. The other four had generic research nurses who covered multiple studies across the hospital. The 7 low-recruiting ICUs reported very infrequent or no use of BAL in the usual diagnosis of suspected VAP. Five reported routine use of empirical antibiotics on suspicion of VAP. The other two ICUs reported routine practice as waiting for confirmation from cultures before commencing antibiotics (if the patient remained stable) and typically completing a prescribed course of antibiotics for the duration, irrespective of clinical changes.

There was no evidence of trial champions in the 7 low-recruiting units. Routine availability of the PI or other clinicians was reported to be low, as was availability of someone to perform BAL. Involvement by clinicians outside of the research team was considered low.

High staff turnover combined with frequent changes in junior doctors' rotas were felt to contribute to attrition of trial visibility and expertise. Recruitment slowed in the last year of the trial, which was felt to have impacted negatively on motivation within research teams.

PIs at the seven low-recruiting ICUs were perceived to prefer mini-BAL outside of the trial, and use of mini-BAL was considered to have prevented recruitment of some patients. The low-recruiting ICUs also had a strong culture of not de-escalating antibiotics, on the basis of perceived risk, thereby reducing recruitment. A risk-averse culture was described in these ICUs.

Occasional lack of available technicians to process BAL samples at the appropriate times, coupled with cut-off times for receipt of samples imposed by laboratories, accounted for missing 20% of eligible patients within the low-recruiting group. Finally, antibiotic recommendations from the laboratory did not always reach a clinician who had been involved in recruitment of the patient. Due to shift changes, recommendations were often fed back to someone outside the research team (commonly during the evening), who may have been unfamiliar with the study requirements.

Reasons for non-compliance with trial recommendations to discontinue antibiotics

During the trial there were 17 recommendations to discontinue antibiotics in the biomarker-guided group, four of which were complied with. Reasons for non-compliance, determined from the process evaluation, are described in Table S37.

Questionnaire completed?	Stated indication for antibiotics and/or non-compliance
Yes	Patient septic due to multiple causes<br>*Confidence in biomarker:* 3/4<br>*Reason for score:* Has to be statistically proven.
Yes	Blood cultures identified Gram-positive rods. *Staphylococcus aureus* grown in NBL. Microbiology review suggested continuing flucloxacillin as appropriate treatment.<br>*Confidence in biomarker:* 1/4<br>*Reason for score:* In light of the above the biomarker result would be of secondary importance given the clinical situation (pyrexia, increased sputum load & signs of left lower lobe consolidation).
Yes	Result from a BAL done 4 days before recruitment came back positive for *Klebsiella*.<br>*Confidence in biomarker:* 3/4<br>*Reason for score:* 1st time of use. Would have acted on it if positive culture from recent BAL had not come back.
Yes	*Pseudomonas* and *Escherichia coli* grown from abdominal fluid. Therefore, decision to resume antibiotics. No concern over chest as source of sepsis.<br>*Confidence in biomarker:* 4/4<br>*Reason for score:* Antibiotics given for intra-abdominal sepsis
No	VAP
No	VAP
No	HAP
No	VAP
No	VAP
No	HAP
No	+ve culture
No	VAP
No	No data available

Table S36: Reasons for non-compliance with recommendation to discontinue antibiotics.

Health resource utilisation

The estimated mean cost of hospital service use was £31,042 (95% CI 26,651 – 35, 433) and £30,750 (95% CI 26,616 – 34,885) for the intervention and the control arms, respectively, in the ITT population (p=0·92).
